# Unique Structure and Dynamics of the EphA5 Ligand Binding Domain Mediate Its Binding Specificity as Revealed by X-ray Crystallography, NMR and MD Simulations

**DOI:** 10.1371/journal.pone.0074040

**Published:** 2013-09-24

**Authors:** Xuelu Huan, Jiahai Shi, Liangzhong Lim, Sayantan Mitra, Wanlong Zhu, Haina Qin, Elena B. Pasquale, Jianxing Song

**Affiliations:** 1 NUS Graduate School for Integrative Sciences and Engineering, National University of Singapore, Singapore, Republic of Singapore; 2 Department of Biological Sciences, Faculty of Science, National University of Singapore, Singapore, Republic of Singapore; 3 Sanford-Burnham Medical Research Institute, La Jolla, California, United States of America; 4 Pathology Department, University of California San Diego, La Jolla, California, United States of America; 5 Department of Biochemistry, Yong Loo Lin School of Medicine, National University of Singapore, Singapore, Republic of Singapore; Rutgers University, United States of America

## Abstract

The 16 EphA and EphB receptors represent the largest family of receptor tyrosine kinases, and their interactions with 9 ephrin-A and ephrin-B ligands initiate bidirectional signals controlling many physiological and pathological processes. Most interactions occur between receptor and ephrins of the same class, and only EphA4 can bind all A and B ephrins. To understand the structural and dynamic principles that enable Eph receptors to utilize the same jellyroll β-sandwich fold to bind ephrins, the VAPB-MSP domain, peptides and small molecules, we have used crystallography, NMR and molecular dynamics (MD) simulations to determine the first structure and dynamics of the EphA5 ligand-binding domain (LBD), which only binds ephrin-A ligands. Unexpectedly, despite being unbound, the high affinity ephrin-binding pocket of EphA5 resembles that of other Eph receptors bound to ephrins, with a helical conformation over the J–K loop and an open pocket. The openness of the pocket is further supported by NMR hydrogen/deuterium exchange data and MD simulations. Additionally, the EphA5 LBD undergoes significant picosecond-nanosecond conformational exchanges over the loops, as revealed by NMR and MD simulations, but lacks global conformational exchanges on the microsecond-millisecond time scale. This is markedly different from the EphA4 LBD, which shares 74% sequence identity and 87% homology. Consequently, the unbound EphA5 LBD appears to comprise an ensemble of open conformations that have only small variations over the loops and appear ready to bind ephrin-A ligands. These findings show how two proteins with high sequence homology and structural similarity are still able to achieve distinctive binding specificities through different dynamics, which may represent a general mechanism whereby the same protein fold can serve for different functions. Our findings also suggest that a promising strategy to design agonists/antagonists with high affinity and selectivity might be to target specific dynamic states of the Eph receptor LBDs.

## Introduction

The Eph receptors constitute the largest family of receptor tyrosine kinases, with 16 members in vertebrates, which can be activated by 9 ephrin ligands [1]–[6]. Eph receptors and ephrins are both anchored onto the plasma membrane, and the interactions between them initiate bidirectional signals that direct pattern formation and morphogenetic processes such as axon growth, cell assembly and migration, and angiogenesis [1]–[8]. As they function in both physiology and disease, Eph receptors and ephrins also represent promising targets for drug design.

Based on their sequence conservation and binding preferences, Eph receptors and ephrins are subdivided into two classes: A and B. In general, EphA receptors (EphA1-A10) only interact with glycosylphosphatidylinositol (GPI)-anchored ephrin-A ligands (ephrin-A1-A6), while EphB receptors (EphB1-B6) interact with transmembrane ephrin-B ligands (ephrin-B1-ephrin-B3). Interactions between the Eph receptors and ephrins of the same class are quite promiscuous but interactions between classes are relatively rare. EphA4 is the only receptor capable of interacting with all 9 ephrins of both A and B classes [7].

All Eph receptors share the same modular structure, consisting of a unique N-terminal globular domain that mediates high-affinity ephrin binding [8], [9], followed by a cysteine-rich linker and two fibronectin type III repeats in the extracellular region. The intracellular region is composed of a juxtamembrane segment, a conserved tyrosine kinase domain, a C-terminal sterile α-domain, and a PDZ domain-binding motif. The crystal structures of several Eph receptor ligand-binding domains (LBDs) in the free state or in complex with ephrins or peptides have been determined [8]–[21]. The LBDs of both EphA and EphB receptors adopt the same jellyroll β-sandwich architecture composed of 11 antiparallel β-strands connected by loops of various lengths. The formation of a complex between an Eph receptor and an ephrin is characterized by the insertion of the solvent exposed ephrin G–H loop into an Eph receptor hydrophobic pocket delimited by a convex sheet of four β-strands capped by the D–E, G–H and J–K loops. A notable feature uncovered by the previously determined structures is that while the jellyroll β-sandwich core is highly similar regardless of whether an Eph receptor is unbound or in complex with a ligand, loops such as the D–E, G–H and J–K loops can adopt dramatically different conformations.

The unique ability of the Eph receptor LBD to use the same fold to bind ephrins, antagonistic peptides, small molecules [12], [22], [23] and the MSP domain of VAPB [24], [25] makes them an attractive model system for deciphering the structural and dynamic principles governing protein-ligand interactions. Our understanding of molecular recognition is still incomplete and the role of protein dynamics in the regulation of binding affinity and specificity is only beginning to be understood [14], [25]–[36]. We previously used crystallography, NMR spectroscopy and molecular dynamics simulations to demonstrate the co-existence of multiple conformations over the loops of the unbound EphA4 LBD, which can interconvert on the picosecond to nanosecond (ps-ns) time scale [14]. Moreover, the high-affinity ephrin-binding pocket of the EphA4 LBD appears to also undergo a significant conformational transition from a closed to an open state in order to bind ephrins [14]. This conformational exchange is characterized by the chameleon transformation of a short β-sheet in the J–K loop into helical-like conformations. Molecular dynamics simulations imply that the closed and open conformations are separated by a relatively large energy barrier and consequently their interconversion likely occurs on the microsecond to millisecond (µs-ms) time scale. Therefore, open and closed conformational states of the EphA4 LBD that can bind diverse ligands already co-exist despite being unbound, and the introduction of a certain ligand shifts the equilibrium towards an increase in the conformational state that binds that ligand [14], [35]. Consistent with this scenario, using NMR spectroscopy, we recently found that the EphA4 LBD undergoes dramatic conformational exchanges not only in loop regions on ps-ns, but also over the whole molecule on µs-ms time scales [36].

To assess whether protein dynamics also play a key role in the binding of other Eph receptors to their ephrin ligands, we have determined the crystal structure of the unbound EphA5 LBD and characterized its dynamics on three time scales using NMR spectroscopy and molecular dynamics simulations. EphA5 binds only A-class ephrins [37] and is highly expressed in the developing nervous system, where it plays important roles in repulsive axon guidance and synaptogenesis [38]–[40]. EphA5 is also expressed in many adult tissues, including the adult brain [38]–[42], and has been proposed to play a role in synaptic plasticity and behavior as well as drug addiction [42]–[44]. Outside the nervous system, EphA5 has been found to play a role in insulin secretion in the pancreas [45]. Increased EphA5 expression has also been associated with pancreatic cancer proliferative capacity as well as ovarian and hepatocellular cancer malignancies [46]–[49]. However, other studies have shown that the EphA5 promoter is hypermethylated in colorectal and breast cancer leading to decreased receptor expression [50], [51], and that EphA5 expression is associated with tumor dormancy [52].

Our study reveals that the EphA5 LBD has dynamic properties very different from those of the EphA4 LBD, despite the 74% sequence identity (87% homology), their structural similarity and their shared ability to bind ephrin-A ligands. This supports the notion that the global conformational exchanges of the EphA4 LBD on the µs-ms time scale play a key role in its ability to also bind ephrin-B ligands.

## Materials and Methods

### Accession Numbers

The structure coordinates of the EphA5 LBD was deposited in Protein Data Bank, with RCSB ID code of rcsb072038 and PDB ID code of 4ET7.

### Cloning, expression and purification of the EphA5 ligand-binding domain

The DNA fragment encoding the human EphA5 ligand-binding domain (LBD), including residues 59–235 (GenBank accession number: AAI43428.1) was amplified from a HeLa cell cDNA library by using two primers containing BamHI and XhoI restriction sites, 5′-GGA TCC AAC GAA GTG AAT TTA TTG GAT TCA CGC -3′ (forward) and 5′-CTC GAG TCA AGA AGG CGC TTC TTT ATA GTA TAC -3′ (reverse). The PCR fragment was cloned into a modified pET32a vector (Novagen), and subsequently the vector was transformed into *E. coli* Rosetta-gami (DE3) cells (Novagen) as we previously performed for the EphA4 LBD [14], [17], which allows more efficient formation of disulfide bonds and expression of eukaryotic proteins containing codons rarely used in *E. coli*.

The recombinant EphA5 LBD was over-expressed by culturing *E. coli* Rosetta-gami cells in Luria-Bertani medium at 37°C until the absorbance at 600 nm reached ∼0.6. Then 0.1 mM isopropyl 1-thio-D-galactopyranoside (IPTG) was added to induce the over-expression at 18°C overnight. The harvested cells were sonicated in lysis buffer containing 150 mM sodium chloride, 20 mM sodium phosphate (pH 7.3) to release soluble His-tagged EphA5 LBD protein, which was subsequently purified by affinity chromatography using nickel-nitrilotriacetic acid-agarose (Qiagen). In-gel cleavage of the fusion protein to release the EphA5 LBD was performed at room temperature by incubating the fusion protein attached to nickel-nitrilotriacetic acid-agarose with thrombin overnight. The released EphA5 LBD was first purified with an AKTA FPLC machine (Amersham Biosciences) on a gel filtration column (HiLoad 16/60 Superdex 200) equilibrated with a buffer containing 150 mM sodium chloride, 20 mM sodium phosphate at pH 7.3, followed by a purification on an anion-exchange column (Mono Q 5/50) with a gradient of 0–1 M NaCl in 25 mM Tris-HCl buffer, pH 7.8.

### Preparation of the WDC antagonistic peptide

To obtain the antagonistic peptide WDCNGPYCHWLG (WDC), a PCR-based strategy was utilized to synthesize its gene with *E. coli* preferred codons. Briefly, the gene was obtained by the PCR reaction of two long oligonucleotides: Forward Primer (5′-GGA TCC TGG GAT TGC AAC GGC CCG TAT TGC CAT TG -3′) and Reverse Primer (5′-CTC GAG TCA GCC CAG CCA ATG GCA ATA CGG GCC-3′) with a 17-mer overlap containing BamHI and XhoI restriction sites. The PCR fragment was subsequently cloned into a modified pGEX-4T-1 vector (Amersham Biosciences), which was transformed into *E. coli* Rosetta-gami (DE3) cells for the expression (Novagen). The peptide was released from the GST fusion protein by in-gel thrombin cleavage followed by HPLC purifications on a RP-18 column (Vydac). The identity of the recombinant peptide was verified by electrospray mass spectrometry and NMR resonance assignments, which showed that the intramolecular disulfide was already formed.

A modified WDC peptide (WDCNGPYCHWLG-(PEG)_2_-KK) was synthesized by Anaspec, Inc. (San Jose, CA) and induced to form an intramolecular disulfide bond between the two cysteine residues. The purity of peptide with the intramolecular disulfide bond was determined to be 99% by analytical HPLC.

### Structure determination of the EphA5 LBD

The EphA5 LBD was prepared in a buffer containing 25 mM Tris-HCl (pH 7.5), 150 mM NaCl and 5 mM CaCl_2_ at a concentration of 10 mg/ml. A crystal screen was set up by preparing 1 μl of the protein solution mixed with 1μl of the reservoir solution as hanging drops at room temperature in a well containing the reservoir solution. Rock-like crystals formed in the well containing 0.1 M Tris-HCl (pH 8.5) and 2.0 M ammonium sulfate after 5 days.

The crystal was protected by cryoprotectant (0.1 M Tris-HCl, 2.0 M ammonium sulfate, 25% glycol, pH 8.5). X-ray diffraction images for a single crystal were collected using an in-house Bruker X8 PROTEUM x-ray generator with a CCD detector. The data were indexed and scaled by HKL2000 package [53] to be in the space group C222_1_ (a = 55.04, b = 82.72, c = 81.17), with one molecule per asymmetric unit, using the program SAINT. The Matthews coefficient was 2.27 with 45.94% solvent constant by CCP4 software package [54]. All figures were prepared using the PyMOL molecular graphics system (Delano Scientific LLC, San Carlos, CA).

### ITC and NMR characterization of the binding of an antagonistic peptide and an antagonistic small molecule to the EphA5 LBD

ITC experiments were performed using a Microcal VP ITC machine as we previously conducted on the EphA4 LBD [12], [17]. Titrations of bacterially expressed WDC binding to the EphA5 LBD were conducted in 10 mM phosphate buffer, pH 6.3, at 25°C. The EphA5 LBD was placed in a 1.8 mL sample cell, while the WDC peptide was loaded into a 300 μL syringe. The samples were degassed for 15 min to remove bubbles before titrations were initiated. A control titration experiment with the same parameters setting was also performed without the EphA5 LBD to measure the contribution of peptide dilution. To obtain thermodynamic binding parameters, the titration data after subtracting the values obtained from the control experiments were fitted to a single binding site model using the built-in software ORIGIN version 5.0 (Microcal Software Inc.).

NMR samples were prepared in 10 mM phosphate buffer, pH 6.3, with the addition of 10% D_2_O for NMR spin-lock. All NMR data were collected at 25°C on an 800-MHz Bruker Avance spectrometer equipped with a shielded cryoprobe as described previously [12], [14]. For the sequential assignment, a pair of triple-resonance NMR spectra, HNCACB and CBCA(CO)NH, were acquired on a double-labeled EphA5 sample at a concentration of 800 µM.

For NMR characterization of the binding of the EphA5 LBD with the WDC peptide and the small molecule C1, two-dimensional ^1^H-^15^N HSQC spectra were acquired at a protein concentration of 100 µM in the absence and in the presence of the WDC peptide or C1 at different molar ratios.

### IC_50_ determination for the inhibition of ephrin-A5 binding to EphA5 by the WDC antagonistic peptide

Protein A-coated wells (Pierce Biotechnology, Rockford, IL) were incubated with a 50 µL solution of 1 µg/mL rat EphA5 Fc in Tris buffer (150 mM NaCl, 50 mM Tris-HCl, pH 7.5) containing 0.02 mM Tween-20 for 1 hour. Wells coated with rat EphA5 Fc (R&D Systems) were then rinsed with Tris buffer, 0.01% Tween 20, and incubated with different synthetic WDC peptide concentrations and 0.6 nM ephrin-A5 AP (0.94 OD min^−1^ mL^−1^) in a total volume of 50 µL for 1 hour. After washing away the unbound peptide and ephrin, bound ephrin-A5 AP was detected using 1 mM pNPP substrate. Data were fitted using non linear regression and IC_50_ values were calculated using the program Prism (GraphPad Software Inc.).

### Eph receptor selectivity of the WDC antagonistic peptide

Protein A-coated wells (Pierce Biotechnology, Rockford, IL) were incubated at room temperature with 50 µL solutions of 1 µg/mL EphA Fc receptors (EphA2-EphA8; R&D Systems) and EphB Fc receptors (EphB1-EphB4 and EphB6) in Tris buffer for 1 hour. The wells were then rinsed with Tris buffer, 0.01% Tween 20 and incubated for 1 hour with either 0.6 nM ephrin-A5 AP (0.94 OD min^−1^ mL^−1^) for the EphA receptors, or 0.2 nM ephrin-B2 AP (0.32 OD min^−1^ mL^−1^) for the EphB receptors in the presence or absence of 100 µM synthetic WDC peptide. After washing away unbound ephrin and peptide, the amount of bound ephrin was detected using 1 mM pNPP substrate.

### Pulsed gradient field NMR determination

Pulsed gradient field (PGF) NMR experiments were utilized to assess the oligomerization properties [55] of the EphA5 LBD in 10 mM phosphate buffer at pH 6.3, which were acquired at 25°C on an 800 MHz Bruker Avance spectrometer. The NMR experiments were implemented by use of the Bruker pulse sequence ledbpgpprwg2 s and the Bruker macro diffusion ordered spectroscopy (DOSY). Typically 16 values of gradient strength were used in the range 0 to 32 G/cm, with PFG duration of 2 ms, and diffusion time of 150 ms. The self-diffusion coefficients (*D_s_*) were calculated using the Bruker DOSY analysis program, with intensities of peaks at −0.057 and −0.177 ppm. Each sample was run in triplicate and *Ds* values were averaged over the three experiments.

### Protein dynamics on the second-hour time scale as studied by NMR spectroscopy

Hydrogen-deuterium (H/D) exchange experiments were conducted on the EphA5 LBD to gain an initial insight into its dynamic behavior on the ms-hr time scale, as we previously described for human ephrin-B2 and EphA4 [12]. Briefly, the ^15^N-labeled EphA5 LBD in the 10 mM, pH 6.3 phosphate buffer was lyophilized and then re-dissolved in D_2_O. The progress of the exchange process between amide protons and deuterium was followed by collecting a series of successive HSQC spectra starting immediately after sample re-solubilization in D_2_O. All exchange experiments were conducted on an 800 MHz Bruker Avance spectrometer at 25°C. The first HSQC spectrum was collected after 15 min, and the last spectrum was acquired after 24 hours.

### Protein dynamics on ps-ns time scale as studied by NMR spectroscopy

^15^N backbone T1 and T1ρ relaxation times and {^1^H}-^15^N steady state NOE intensities were collected on an 800 MHz Bruker Avance spectrometer equipped with both an actively shielded cryoprobe and pulse field gradient units [56]. Relaxation time T1 was determined by collecting 8 points with delays of 10, 250, 650, 900, 1000, 1100, 1300 and 1400 ms using a recycle delay of 1 s, with a repeat at 650 ms. Relaxation time T1ρ was measured by collecting seven points with delays of 1, 22, 35, 48, 60, 70, 76 ms using a spin-lock power of 1.6 kHz and a 2.5 s recycle delay with a repeat at 48 ms. {^1^H}-^15^N steady-state NOEs were obtained by recording spectra with and without ^1^H presaturation, a duration of 3 s and a relaxation delay of 6 s at 800 MHz. Relaxation times were fitted to peak height data as single exponential decays.

### Model-free analysis

NMR relaxation data were analyzed by “Model-Free” formalism with protein dynamics software DYNAMICS [57]. Briefly, relaxation of protonated heteronuclei is dominated by the dipolar interaction with the directly attached ^1^H spin and by the chemical shift anisotropy mechanism [56]–[59]. Relaxation parameters are given by:
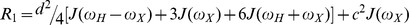





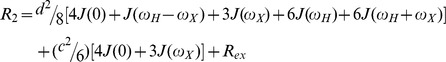


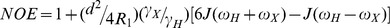



In which, 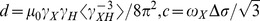, 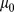
is the permeability of free space; 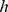 is Planck's constant; 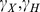
are the gyromagnetic ratios of ^1^H and the X spin (X = ^13^C or ^15^N) respectively; 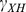 is the X-H bond length; 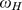
and 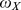
are the Larmor frequencies of ^1^H and X spins, respectively; and
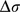
is the chemical shift anisotropy of the X spin.

The Model-Free formalism, as previously established [58] and further extended [59], determines the amplitudes and time scales of the intramolecular motions by modeling the spectral density function, *J*(*ω*), as
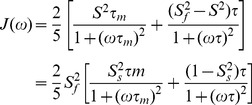
In which, 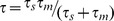, 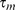
is the isotropic rotational correlation time of the molecule, 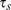 is the effective correlation time for internal motions, 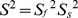 is the square of the generalized order parameter characterizing the amplitude of the internal motions, and 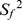
and 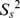
are the squares of the order parameters for the internal motions on the fast and slow time scales, respectively.

In order to allow for diverse protein dynamics, several forms of the spectral density function, based on various models of the local motion [56]–[59], were utilized, which include the original Lipari-Szabo approach, assuming fast local motion characterized by the parameters *S* ^2^ and *τ_loc_*; extended model-free treatment, including both fast (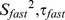) and slow (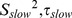) reorientations for the NH bond (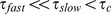); and could also allow for slow, milli- to microsecond dynamics resulting in a conformational exchange contribution, *R_ex_*, to the linewidth.

### Protein dynamics on µs-ms time scale as studied by NMR spectroscopy

^15^N transverse relaxation dispersion experiments for EphA5 LBD in the free state were acquired on a Bruker Avance 800 spectrometer equipped with a z-axis gradient cryoprobe at 298 K [60]. A constant time delay (*T*_CP_  = 50 ms) was used with a series of CPMG frequencies, ranging from 40 Hz, 80 Hz, 120 Hz (x2), 160 Hz, 200 Hz, 240 Hz, 320 Hz, 400 Hz, 480 Hz, 560 Hz, 640 Hz, 720 Hz, 800 Hz, and 960 Hz (x2 indicates repetition). A reference spectrum without the CPMG block was acquired to calculate the effective transverse relaxation rate by the following equation:
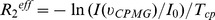
Where I(ν_CPMG_) is the peak intensity on the difference CPMG frequency, I_0_ is the peak intensity in the reference spectrum.

### Molecular dynamics simulations

To unravel the intrinsic dynamics of the EphA5 LBD, three independent, 30 ns molecular dynamics simulations were performed as we previously conducted [14], [25], [29]. Briefly, the simulation cell is a periodic cubic box with a minimum distance of 9 Å between the protein and the box walls to ensure the proteins does not directly interact with its own periodic image. The water molecules, described using the TIP3P model, were filled in the periodic cubic box for the all atom simulations. Each set of molecular dynamics simulations was implemented by using the program GROMACS [61] for 30 ns, with the AMBER 99SB-ILDN all-atom force field. The long-range electrostatic interactions were treated using the fast particle-mesh Ewald summation method. The temperature during simulations was kept constant at 300 K by Berendsen's coupling. The pressure was held at 1 bar. The isothermal compressibility was 4.6*10^−5^ bar^−1^. The time step was set as 2 fs. All bond lengths including hydrogen atoms were constrained by the LINCS algorithm. Prior to molecular dynamics simulations, the initial EphA5 LBD structure was relaxed by 5,000 steps of energy minimization using a steepest descent algorithm, followed by position restraint equilibration for 200 ps.

## Results

### Unique crystal structure of the EphA5 LBD

The human EphA5 LBD was cloned and expressed in soluble form in *E. coli.* In solution this domain is a monomer, as assessed by FPLC size exclusion chromatography (data not shown). We also used pulsed gradient field NMR to measure the self-diffusion coefficient of the EphA5 LBD, which is an indicator of aggregation state in solution because it depends on the radius of a globular protein. The self-diffusion coefficient is 1.10×10^−10^±1.87×10^−12^ m^2^/s at a concentration of 500 µM, which is very similar to that of the monomeric EphA4 LBD (1.08×10^−10^±2.36×10^−12^ m^2^/s) [11]–[13], implying that the EphA5 LBD is also a monomer.

**Figure 1:**
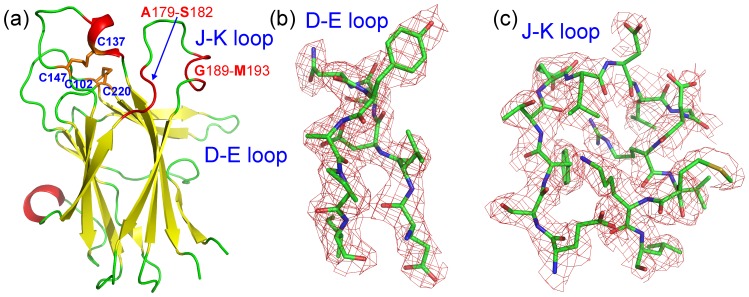
The crystal structure of the EphA5 LBD shows well defined D–E and J–K loops. (a) Crystal structure of the EphA5 LBD. Residues Ala179-Ser182 and Gly189-M193 in the J–K loop, which adopt unusual helical-like conformations, are displayed in red. Two disulfide bridges Cys137-Cys147 and Cys102-Cys220, are displayed in orange. (b, c) Electron density maps for the D–E and J–K loops, showing that all residues are well defined.

The crystal structure of the EphA5 LBD was determined at 2.6 Å resolution by the molecular replacement method using the EphA4 LBD (3CKH) as a search module (Table S1). In the crystal structure all residues are visible except for the last three (Figure 1a) and one asymmetric unit contains only one EphA5 molecule, which does not show any close contacts with other EphA5 molecules in the neighboring units. Overall, the EphA5 LBD adopts the conserved jellyroll architecture characteristic of other Eph receptors, composed of 11 antiparallel β-sheets arranged as a compact β-sandwich. There are two conserved disulfide bridges, one within the G–H loop (Cys137–Cys147) and the other between the E–F and L–M loops (Cys102–Cys220).

**Figure 2:**
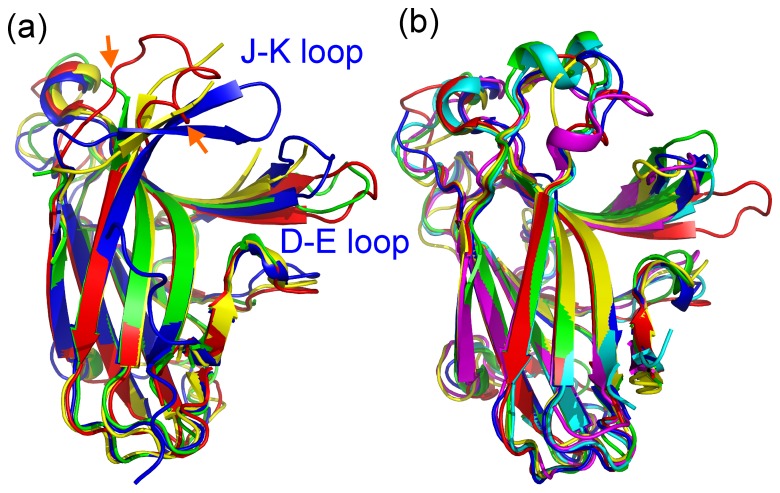
The structure of the unbound EphA5 LBD resembles that of other Eph receptors bound to ephrin ligands. (a) Superimposition of the LBD structures of unbound EphA5 (red), EphA2 (green, 3C8X), EphA4 (yellow, 3CKH) and EphB2 (blue, 3ETP). A short β-sheet is formed by the EphA4 and EphB2 residues corresponding to EphA5 residues Ala179-Ser182 and Gly189-M193 in the J–K loop (orange arrows). (b) Superimposition of the LBD structures of the unbound EphA5 (red), EphA2 in complex with ephrin-A2 (green, 3CZU), EphA4 with ephrin-A2 (cyan, 3WO3), EphA4 with ephrin-B2 (blue, 3GXU), EphB2 with ephrin-B2 (pink, 1KGY) and EphB4 with ephrin-B2 (yellow, 2HLE).

The D–E and J–K loops, which cap the high affinity ephrin-binding pocket, are well-defined in the crystal structure, with high quality electronic densities (Figure 1b, c). This is striking because in previously determined unbound Eph receptor LBD structures, except that of EphA2, the D–E and J–K loops are either entirely or partially invisible due to their high intrinsic dynamics (Figure 2a). Remarkably, in the unbound EphA5 structure Ala179-Ser182 and Gly189-Met193 in the J–K loop adopt a helical-like conformation that resembles the structures previously observed for ephrin-bound Eph receptors (Figure 2b). In contrast, in the unbound EphB2 and EphA4 LBDs the corresponding J–K loop residues form a short antiparallel β-sheet (Figure 2a). Moreover, in the EphA5 LBD structure the distance between D–E and J–K loops is larger than in other unbound Eph receptor LBD structures. Hence, the high affinity ephrin binding pocket of the EphA5 LBD resembles the open conformations previously observed only in Eph receptor LBDs bound to ephrins (Figure 2b).

### The EphA5 LBD is capable of unique ligand binding specificity

**Figure 3:**
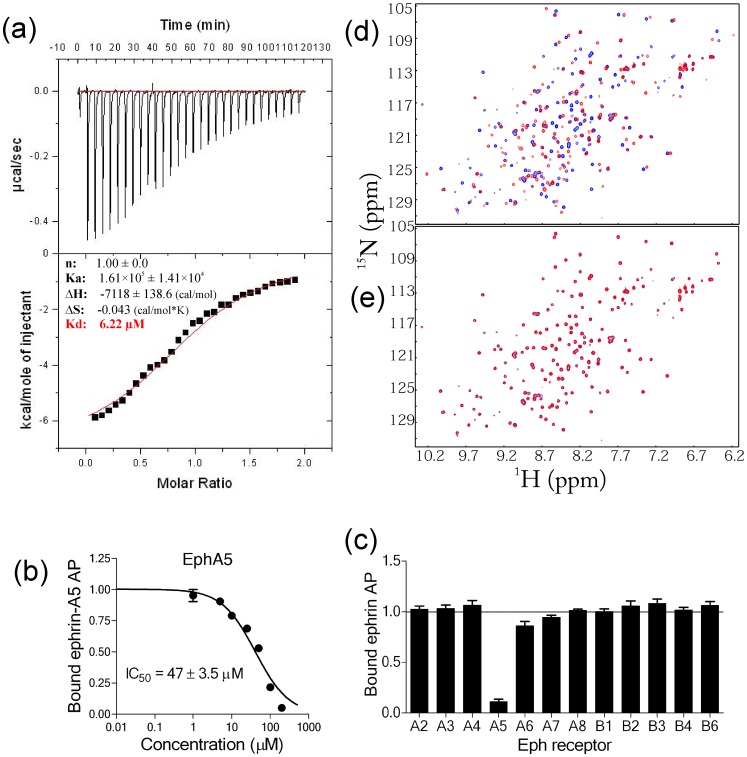
Unique ligand-binding specificity of the EphA5 LBD. (a) Isothermal titration calorimetry profiles for the interaction of the EphA5 LBD with the WDC peptide (upper panel) and plots of the integrated values for the reaction heats (after blank subtraction and normalization to the amount of the peptide injected) versus EphA5 to WDC molar ratio (lower panel). The thermodynamic binding parameters are shown in the lower panel. (b) Inhibition of ephrin-A5 alkaline phosphatase (AP) binding to immobilized EphA5 Fc by increasing concentrations of WDC in ELISAs. Bound ephrin-A5 AP represents the ratio of the OD at 405 nm for ephrin-A5 AP bound to EphA5 Fc in the presence of the indicated concentrations of the WDC peptide and in the absence of peptide. (c) Inhibition of ephrin-A5 AP binding to EphA receptors and ephrin-B2 AP binding to EphB receptors by 100 μM WDC. Bound ephrin AP represents the ratio of the OD at 405 nm for ephrin-A5 AP or ephrin-B2 AP bound to different Eph receptor Fc proteins in the presence of WDC peptide and in the absence of peptide. The peptide substantially inhibits ephrin binding only to EphA5. Averages and standard errors from triplicate measurements are shown. (d) Superimposition of the NMR HSQC spectra of the EphA5 LBD in the absence (blue) and in the presence (red) of WDC at a molar ratio of 1:3 (EphA5:WDC). (e) Superimposition of the NMR HSQC spectra of the EphA5 LBD in the absence (blue) and in the presence (red) of C1 at a molar ratio of 1:20 (EphA5:C1).

WDC (WDCNGPYCHWLG) is a 12 amino acid-long peptide that was previously identified in a phage-display screen based on its binding to the entire extracellular domain of rat EphA5 [62]. Isothermal titration calorimetry (ITC) experiments yielded a Kd of 6.2 µM for binding to the EphA5 LBD (Figure 3a), demonstrating that WDC also binds to human EphA5 and targets the LBD. Consistent with this, in ELISA assays the peptide antagonizes EphA5-ephrin-A5 interaction with an IC_50_ value of ∼50 µM (Figure 3b). The synthetic WDC peptide only significantly inhibits ephrin binding to EphA5, but not other Eph receptors (Figure 3c). These results suggest that WDC binds to the high affinity ephrin binding pocket of EphA5, where it competes with ephrins for binding. Moreover, the high selectivity of WDC implies that the high affinity ephrin binding pocket of EphA5 has some unique structural or/and dynamic properties that are not shared by other Eph receptors.

Titrations of the ^15^N-labeled EphA5 LBD with WDC revealed significant shifts of most HSQC peaks of the EphA5 LBD (Figure 3d). This is consistent with previous results with the EphA4-selective KYL peptide, which has a Kd of 0.8 µM, and also induces extensive shifting of the HSQC peaks of the EphA4 LBD [63]. We further probed the uniqueness of the EphA5 ligand binding pocket by using C1, an antagonistic small molecule that shows selectivity for the EphA2 and EphA4 receptors and induces shifts in several HSQC peaks of the EphA4 LBD [12], [64]. Addition of C1 at ratios of up to 1:20 (EphA5:C1) resulted in no detectable shifts of the HSQC peaks (Figure 3e). This demonstrates that the EphA5 LBD does not significantly bind C1, suggesting that the unique structural features of the J–K loop may play a key role in defining its ligand binding specificity.

### The D and E strands of the EphA5 LBD are exposed to the solvent

**Figure 4:**
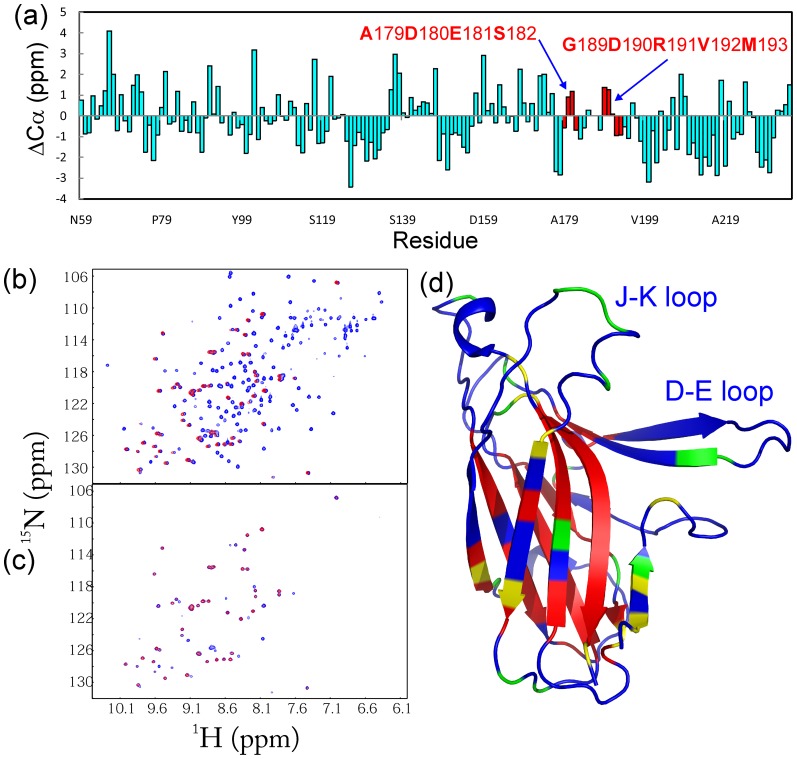
Structural properties and solvent accessibility of the EphA5 LBD in solution. (a) Residue-specific Cα chemical shift deviations (ΔCα  =  δobs – δcoil) for the EphA5 LBD. The bars for the J–K loop residues with helical conformations in the crystal structure are colored in red. (b) Superimposition of ^1^H-^15^N NMR HSQC spectra for the ^15^N-labeled EphA5 LBD at 25°C in 10 mM phosphate buffer, pH 6.5 (blue) and 15 min after dissolving the lyophilized sample in D_2_O (red). The disappearance of the blue HSQC peaks indicates the high exposure of the amide protons to the solvent. (c) Superimposition of HSQC spectra of the EphA5 LBD at 25°C, 15 min (blue) and 24 hours (red) after dissolving the lyophilized sample in D_2_O. (d) EphA5 LBD structure with residues whose HSQC peaks are missing even in H_2_O buffer colored in green, residues whose backbone amide protons completely exchanged within 15 min in blue, residues whose backbone amide protons persisted after 15 min but completely exchanged in 2 hours in yellow, and residues whose backbone amide protons persisted even after 2 hours in red. The very rapid exchange of their amide protons indicates that the D and E strands are highly exposed to the solvent.

To assess the structural and dynamic properties of the EphA5 LBD in solution, we obtained its NMR sequential assignments by analyzing the HNCACB and CBCA(CO)NH pair of triple-resonance NMR spectra. Figure 4a shows residue-specific Cα chemical shift deviations from the random coil values for the EphA5 LBD, which are very sensitive indicators of protein secondary structure. The positive Cα conformational shifts for the J–K loop residues Asp180-Glu181 and Gly189-Asp190 indicate that these two regions adopt a helical-like conformation in solution rather than an extended β-stranded conformation, consistent with the structure of this loop observed in the crystal.

We then utilized NMR hydrogen/deuterium exchange to assess the backbone dynamics of the EphA5 LBD on the min-hr time scale. It is well established that in solution the labile hydrogens of proteins, such as the amide protons, are continually exchanging with the solvent at different rates. The rates of hydrogen exchange depend on a variety of factors associated with the environment, including exposure of the hydrogens to the solvent and their involvement in hydrogen bonds. Consequently, amide hydrogen/deuterium exchange experiments provide a sensitive readout for the degree of exposure of amide protons to the solvent [14]. Approximately ∼58% of the 172 non-proline residues in the EphA5 LBD completely exchanged with deuterium within the experimental dead time of 15 min, indicating that the loop regions are all accessible to the solvent (Figure 4b, d). Interestingly, in addition to the loop residues, which would be predicted to be solvent-exposed, many residues in the D and E β-strands also completely exchanged within 15 min (Figure 4d). This result is dramatically different from our previous finding that most amide protons of residues in the D and E strands of the EphA4 LBD remained unexchanged even after 24 hours [14]. After 2 hours, amide protons of additional EphA5 residues undergo exchange and ∼27% of the residues still have deuterium and can therefore yield HSQC peaks (Figure 4c). These slow-exchanging amide protons are mostly located in the β-strands (Figure 4d). Finally, a few additional HSQC peaks disappear after 24 hours (Figure 4c, d).

### Backbone dynamics of the EphA5 LBD as determined by NMR

**Figure 5:**
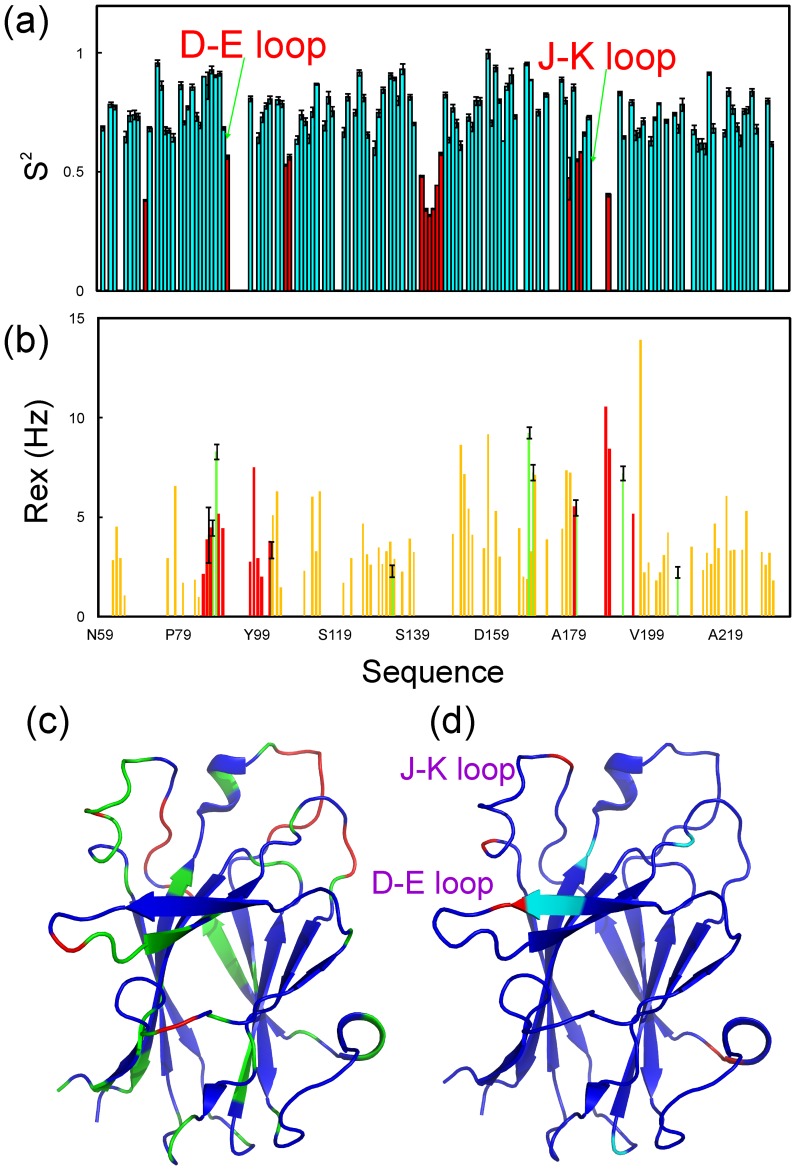
^15^N backbone dynamics for the EphA5 LBD on the ps-ns time scale. (a) Generalized squared order parameter (S^2^) derived from the Model-free analysis of the relaxation data for EphA5. Red indicates residues with S^2^ < the average value. (b) Residue-specific Rex derived from Model-free analysis of relaxation data for EphA5 (green) and EphA4 (red and light brown). Red indicates EphA4 residues in the D and E strands as well as D–E and J–K loops while light brown for the other EphA4 residues. (c) EphA5 LBD structure with residues having S^2^ < the average value (0.7) colored in green and those with S^2^ < the average – STD (0.5) in red. (d) EphA5 LBD structure with residues having Rex >2 Hz colored in cyan and those >5 Hz colored in red.

^15^N NMR backbone relaxation data, including the longitudinal relaxation time T1, transverse relaxation time T2, and {^1^H}-^15^N steady-state NOE are sensitive indicators of protein dynamics on the ps-ns timescale. Figure S1a–c shows the relaxation data for the EphA5 LBD, with the {^1^H}-^15^N steady-state NOEs of most residues forming secondary structures larger than 0.75, which indicates that the regions with secondary structures have significantly limited backbone motion. To gain a quantitative insight, the NMR relaxation data for the EphA5 LBD were further analyzed by “Model-free” formalism [56]–[59]. However, due to the overlap and/or weak intensity of many resonance peaks resulting from the relatively large size of the protein and the presence of many exposed loop residues, only 128 out of 172 non-proline peaks were suitable for this analysis. Isotropic, axially symmetric and fully anisotropic models for the overall motions were examined and compared. A fully anisotropic model was selected, yielding the parameters for the overall rotational diffusion of the free EphA5 LBD shown in Table S2. This analysis generated squared generalized order parameters, S^2^, which reflect the conformational rigidity on a ps-ns time scale. S^2^ values range from 0 for high internal motion to 1 for completely restricted motion in a molecular reference frame. As shown in Figure 5a, the regions with secondary structure have higher S^2^ values, indicating high backbone rigidity, while the loop regions have smaller S^2^ values, suggesting high flexibility. In particular, the residues in the D–E, G–H and J–K loops have relatively low S^2^ values, implying that these residues undergo significant conformational exchanges on the ps-ns time scale.

Model-free analysis also yields Rex values, which reflect conformational exchanges on µs-ms time scale. As shown in Figure 5b, only 10 residues in the EphA5 LBD have Rex values >2 Hz, including residues Gly87-Val89 in the D strand, Lys103 in the E–F loop, Leu134 in the G–H loop, Asn169 and Gln170 in the I–J loop, Glu181 and Met193 in the J–K loop, and Lys207 in the K–L loop (Figure 5b, d). In contrast, Rex values >2 Hz were previously detected over the whole unbound EphA4 LBD [36]. We also conducted CPMG-based relaxation dispersion experiments, but detected only three residues with ΔR_2_ (τ_cp_) >1.5 Hz (Figure S2). Together with the Rex results from the Model-free analysis, this indicates that in the unbound state the EphA5 LBD does not appear to have global conformational exchanges on the µs-ms time scale.

### Dynamics of the EphA5 LBD as determined by molecular dynamics simulations

**Figure 6:**
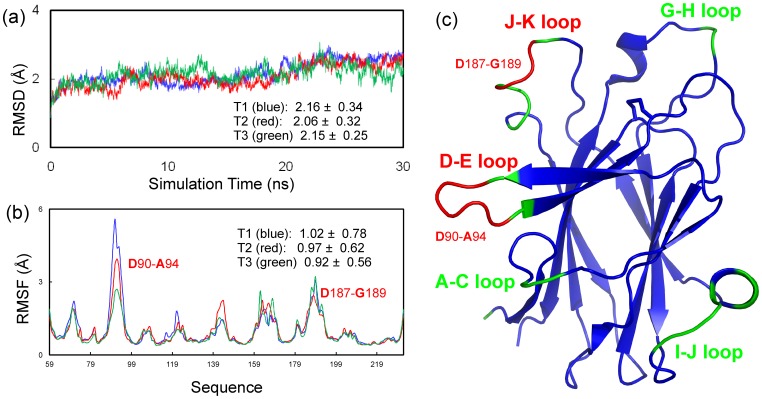
Distinctive dynamic behaviors of the EphA5 LBD as revealed by molecular dynamics simulations. (a) Trajectories of root-mean-square deviations (RMSD) of heavy atoms in three independent molecular dynamics simulations. (b) Trajectories of root-mean-square fluctuations (RMSF) of the Cα atoms computed for three independent simulations, with average values and standard deviations calculated over 30 ns for each simulation. (c) EphA5 LBD structure with the residues having RMSF >average in green and those >2-fold the average in red.

To further explore the dynamic behavior of the EphA5 LBD, we initiated three independent 30 ns molecular dynamics simulations. In all simulations, the root-mean-square deviations (RMSD) values for the heavy atoms increased very rapidly during the first 0.8 ns (Figure 6a). This is mostly due to the relaxations of the crystal structures becoming solvated in solution. Although the three RMSD trajectories have some local differences, their average values over 30 ns are very similar (2.16±0.34, 2.06±0.32 and 2.15±0.25 Å).

Figure 6b shows the root-mean-square fluctuations (RMSF) of the Cα atoms in the EphA5 LBD for the three parallel 30 ns simulations. The average RMSF values over 30 ns are also similar (1.02±0.78, 0.97±0.62 and 0.92±0.56 Å). However, there are some differences in local regions, in particular over D–E loop residues Asp90-Ala94 (Figure 6b), mostly due to the fact that the system behaves as non-ergodic in 30 ns simulations. Interestingly, other than the N-terminus, the residues with RMSF values larger than average are all located over loops, including the A–C, D–E, G–H, I–J, and J–K loops (Figure 6c). Nevertheless, only residues Asp90-Ala94 in the D–E loop and Asp187-Gly189 in the J–K loop have RMSF values larger than 2-fold the average value. These results are consistent with the dynamic behaviors deduced from hydrogen/deuterium exchange (Figure 4) and Model-free analysis (Figure 5).

**Figure 7:**
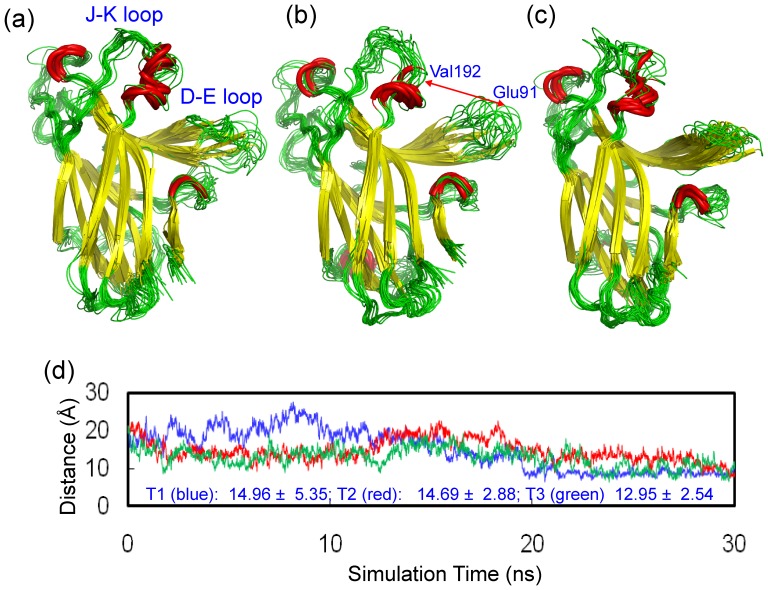
Molecular dynamics simulations reveal that the unbound EphA5 ephrin-binding pocket is in an open conformation. (a–c) Superimposition of structure snapshots taken at 1 ns intervals in three independent molecular dynamics simulations. (d) Trajectories of the distances between the Cα atoms of Glu91 in the D–E loop and Val192 in the J–K loop over 30 ns simulations. The average values and standard deviations calculated over the 30 ns are displayed for each simulation. The EphA5 ligand binding pocket still remains open even after 30 ns in all three simulations.

The extremely high RMSF values for D–E loop residues Asp90-Ala94 represent a unique property of the EphA5 LBD because in molecular dynamics simulations of the EphA4 LBD, the RMSF values of the D–E loop residues were not significantly larger than those of residues in other loops, and in fact were even slightly smaller than those of J–K loop residues [14]. In the unbound EphA4 LBD the D–E loop shows a tendency to move towards the J–K loop (Figure 7), as indicated by the decreasing distances between the Cα atoms of Glu91 in the D–E loop and Val192 in the J–K loop (Figure 7b, d). However even after 20 ns, when this distance becomes largely stable, the average values of this distance still remain very large for EphA5: 8.77±0.84, 12.56±1.76, and 10.76±2.04 Å for the three simulations. It is noteworthy that J–K loop residues Gly189-Met193 have completely different dynamic behaviors in EphA5 and EphA4. In EphA5, these residues initially form a helical-like conformation, which becomes a stable helix in the simulations (Figure 7a–c). In contrast, the corresponding residues form a short β-strand in EphA4, which became further extended in previous molecular dynamics simulations [14].

## Discussion

To understand the structural and dynamic principles that enable different Eph receptors to achieve distinct ligand binding specificities by utilizing the same LBD architecture, in this study we determined the first crystal structure of the LBD of EphA5, a receptor that has higher ligand binding selectivity than the EphA4 LBD. In the EphA5 crystal structure, all D–E and J–K loop residues are visible and adopt helical-like conformations over Ala179-Ser182 and Gly189-Met193. This unique feature was confirmed by NMR characterization. Strikingly, the ligand binding pocket of the unbound EphA5 LBD resembles the open form previously observed only in other Eph receptor LBDs when they are bound to ephrins. That the EphA5 ephrin-binding pocket has an open conformation in the absence of a bound ephrin is strongly supported by the NMR hydrogen-deuterium exchange results showing that most EphA5 D and E strand residues are highly accessible to the solvent. This is markedly different from our previous results showing that most D and E strand residues of the EphA4 LBD are protected from the solvent [14].

**Figure 8:**
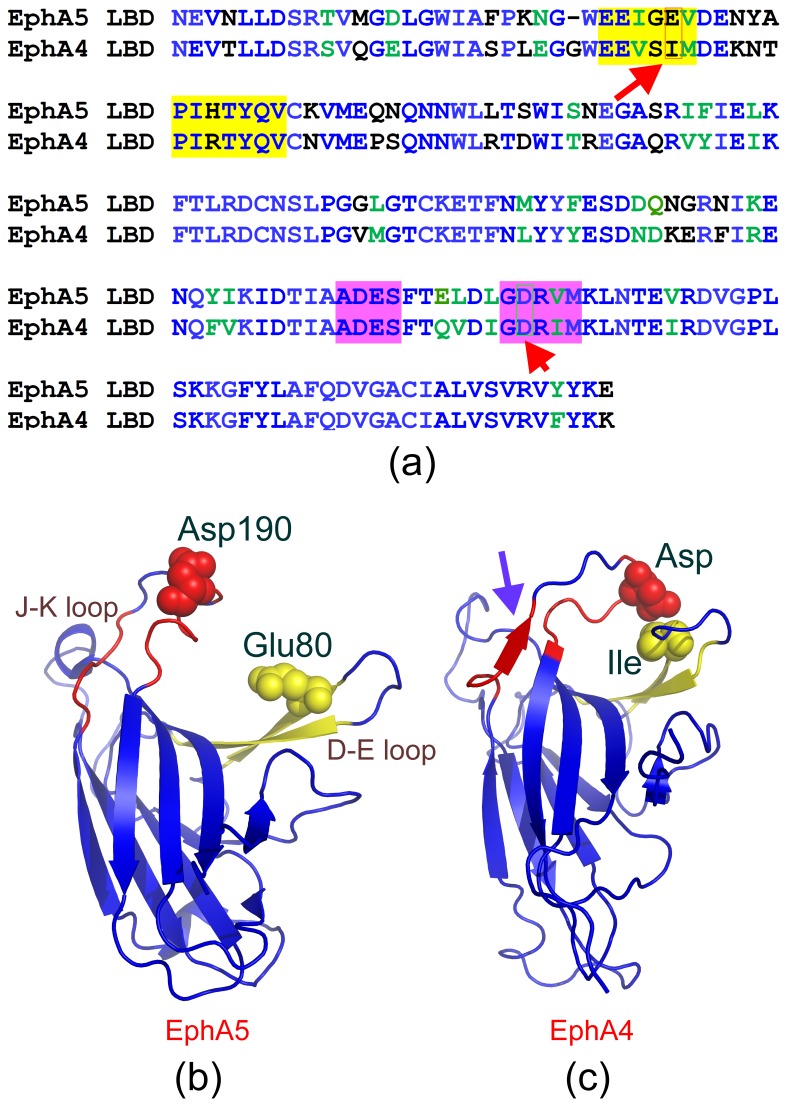
Sequence-structure relationship for the EphA5 and EphA4 LBDs. (a) Alignment of the sequences of the EphA5 and EphA4 LBDs. Identical residues are colored in blue, homologous in green and different in black. Residues in the D and E β-strands are highlighted in yellow and residues in the J–K loop in pink. Two residues that are in close contact in the EphA4 structure (Ile in the D strand and Asp in the J–K-loop) and the corresponding residues in the EphA5 LBD are boxed. (b) Structure of the EphA5 LBD with spheres for Asp190 in the J–K-loop, and Glu80 in the D strand which corresponds to a Ile in the structure of the EphA4 LBD (c).

Surprisingly, although the J–K loop residues Ala179-Ser182 and Gly189-Met193 are identical in EphA5 and EphA4 with the exception of Ile192 in EphA5, which corresponds to the homologous Val in EphA4 (Figure 8a), they assume helical-like conformations in EphA5 (Figure 8b) but form short antiparallel β-sheets in EphA4 (Figure 8c). So what is responsible for this difference? As shown in Figure 8c, the tips of the D–E and J–K loops are in close contact in the EphA4 LBD, with a direct contact of an Ile and an Asp residues. Interestingly, while the EphA4 Asp residue corresponds to Asp190 in EphA5, the EphA4 Ile corresponds to Glu80 in EphA5. Since other D–E and J–K loop residues are either identical in EphA5 and EphA4, or not near the region of interaction between the two loops in the unbound EphA4 structure, it is likely that the repulsive electrostatic force between Glu80 and Asp190 is the main mechanism preventing the D–E and J–K loops of EphA5 from becoming as close as those in the unbound EphA4 LBD structure. This is supported by the molecular dynamics simulation results showing that after 20 ns, when the distance between the D–E and J–K loops of EphA5 has become largely stable, the distance still remains very large as compared with that in the EphA4 LBD [14]. It appears that in EphA5, Ala179-Ser182 and Gly189-Met193 undergo a chameleon transformation into helical-like conformations mostly due to the absence of long-range interactions between D–E and J–K loops. It is well established that helices can be stabilized by local interactions while β-sheets are mostly specified by long-range interactions and their presence is, therefore, highly context-dependent. Consequently, loss of long-range interactions usually leads to the chameleon transformation into helical conformations as exemplified by our previous reports [29], [65], [66].

As the EphA5 ephrin-binding pocket is highly populated with the open conformations, the binding of ephrin-A ligands is expected to trigger a shift in their equilibrium that involves only small variations mostly over the loops instead of the large differences observed for the EphA4 LBD [14]. Since conformations characterized by small differences in the loops are separated by relatively small energy barriers, their exchanges are predicted to occur mostly over the ps-ns time scale. Indeed, our NMR and molecular dynamics results reveal that the EphA5 LBD has intrinsic dynamics in the ps-ns time scale over loop regions, as also previously observed for the EphA4 LBD [14]. However, NMR experiments did not reveal global conformational exchanges on the µs-ms time scale for the EphA5 LBD. This is in contrast to the EphA4 LBD, which has considerable conformational exchanges on the µs-ms time scale over the whole domain [14], [36]. It appears that the open conformations populated by the EphA5 LBD are only suitable for binding ephrin-As, according to the ligand specificity of EphA5.

Our study thus suggests that protein dynamics play a key role in modulating the binding specificity of Eph receptors for various ligands through a conformational selection mechanism [14], [35], as previously observed on other proteins [26]–[28], [30]–[35], [67]–[69]. However, ‘conformational selection’ is a population-based framework and not incompatible with ‘induced fit’ or even ‘lock-and-key’ mechanisms. Indeed, the structural and dynamic properties of the EphA5 LBD enable its unique ligand binding specificity. For example, EphA5 is the only Eph receptor capable of binding WDC with substantial affinity (Kd  = 6 µM). In contrast, C1, an antagonistic small molecule that binds the EphA4 LBD with a Kd of 20 µM [12], [22], shows no detectable binding to EphA5. We previously showed that C1 forms a complex with the EphA4 LBD by interacting with residues on both D–E and J–K loops [12]. Most likely, the inability of C1 to bind to EphA5 is consistent with the idea that the unbound EphA5 LBD is populated with an open conformation in which D–E and J–K loops are not close enough to allow C1 to interact with residues from both loops. However, although our previous study showed that the negatively-charged carboxylic and hydroxyl groups on the benzene ring are involved in hydrogen bonding to the side-chain amide protons of Gln43 in EphA4 [12], it is not possible to exclude that the presence of Glu80 in EphA5 may also contribute to some degree to the lack of C1 binding. These findings imply that a general strategy for the design of selective agonists or antagonists would be to target specific dynamic states of the Eph receptor LBDs, even though they all share a common jellyroll β-sandwich fold.

In conclusion, by the complementary use of crystallography, NMR spectroscopy and computational simulations, we have unraveled the distinctive structural and dynamic properties associated with the EphA5 LBD. Interestingly, our results suggest that although the EphA5 LBD has high sequence homology with the EphA4 LBD and the same overall architecture, it is still able to manifest different binding specificity through conformational selection mechanism as previously proposed [14], [35]. Moreover, our results also imply a mechanism by which differential dynamics of the same three-dimensional fold adopted by highly homologous sequences result in different binding specificities.

## Supporting Information

Figure S1 **^15^N backbone relaxation data for the EphA5 LBD.** (a) R1 values, which are the inverse of T1 (longitudinal) relaxation times. (b) R2 values, which are the inverse of T2 (transverse) relaxation times. (c) {^1^H}-^15^N steady state NOE intensity (hNOE), which offers a reliable measure of backbone dynamics on the ps-ns time scale. (d) EphA5 LBD structure with the residues having hNOE values < the average (0.65) colored in red.
(TIF)

Figure S2 **CPMG dispersion measurements reveal that the EphA5 LBD lacks global conformational exchanges in the µs-ms time scale.** (a) Difference of effective transverse relaxation rate R_2_ (τcp) at 80 and 960 Hz. Only three residues have ΔR_2_ (τ_cp_) >1.5 Hz, which indicates that only these residues have conformational exchanges on the µm-ms time scale. (b) EphA5 LBD with the three residues having ΔR_2_ (τcp) >1.5 Hz displayed as spheres.
(TIF)

Table S1 **Crystallographic data and refinement statistics for the EphA5 LBD structure.**
(DOCX)

Table S2 **Characteristics of the overall rotational diffusion of the EphA5 LBD.**
(DOCX)
