# Supplementary figures and images for: Unique Structure and Dynamics of the EphA5 Ligand Binding Domain Mediate Its Binding Specificity as Revealed by X-ray Crystallography, NMR and MD Simulations
Source: PLoS One. 2013 Sep 24;8(9):e74040. doi: 10.1371/journal.pone.0074040 (PMC3782497; doi:10.1371/journal.pone.0074040)

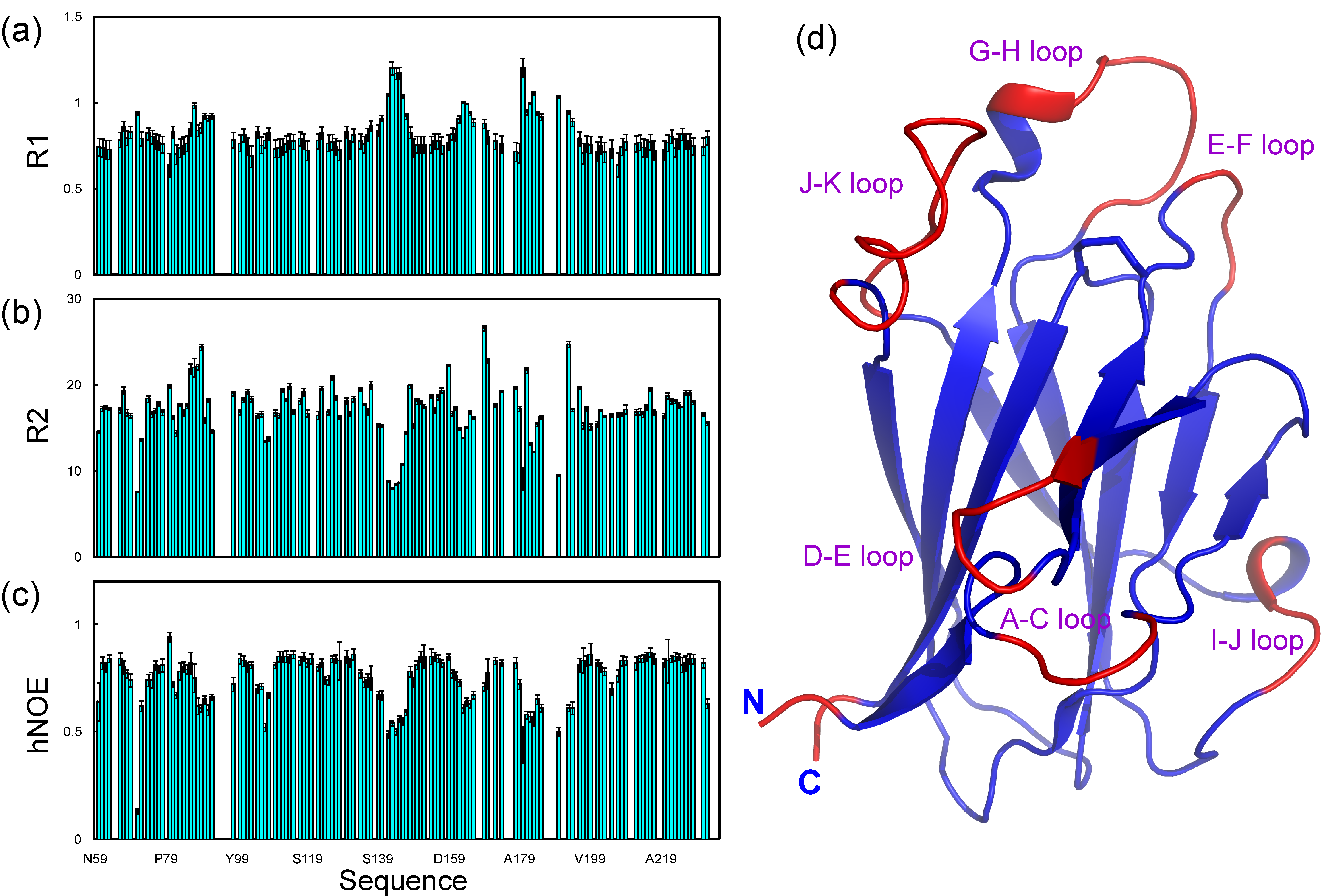

Supplement: Figure S1 — 15N backbone relaxation data for the EphA5 LBD. (a) R1 values, which are the inverse of T1 (longitudinal) relaxation times. (b) R2 values, which are the inverse of T2 (transverse) relaxation times. (c) {1H}-15N steady state NOE intensity (hNOE), which offers a reliable measure of backbone dynamics on the ps-ns time scale. (d) EphA5 LBD structure with the residues having hNOE values < the average (0.65) colored in red. (TIF) [file pone.0074040.s001.tif]

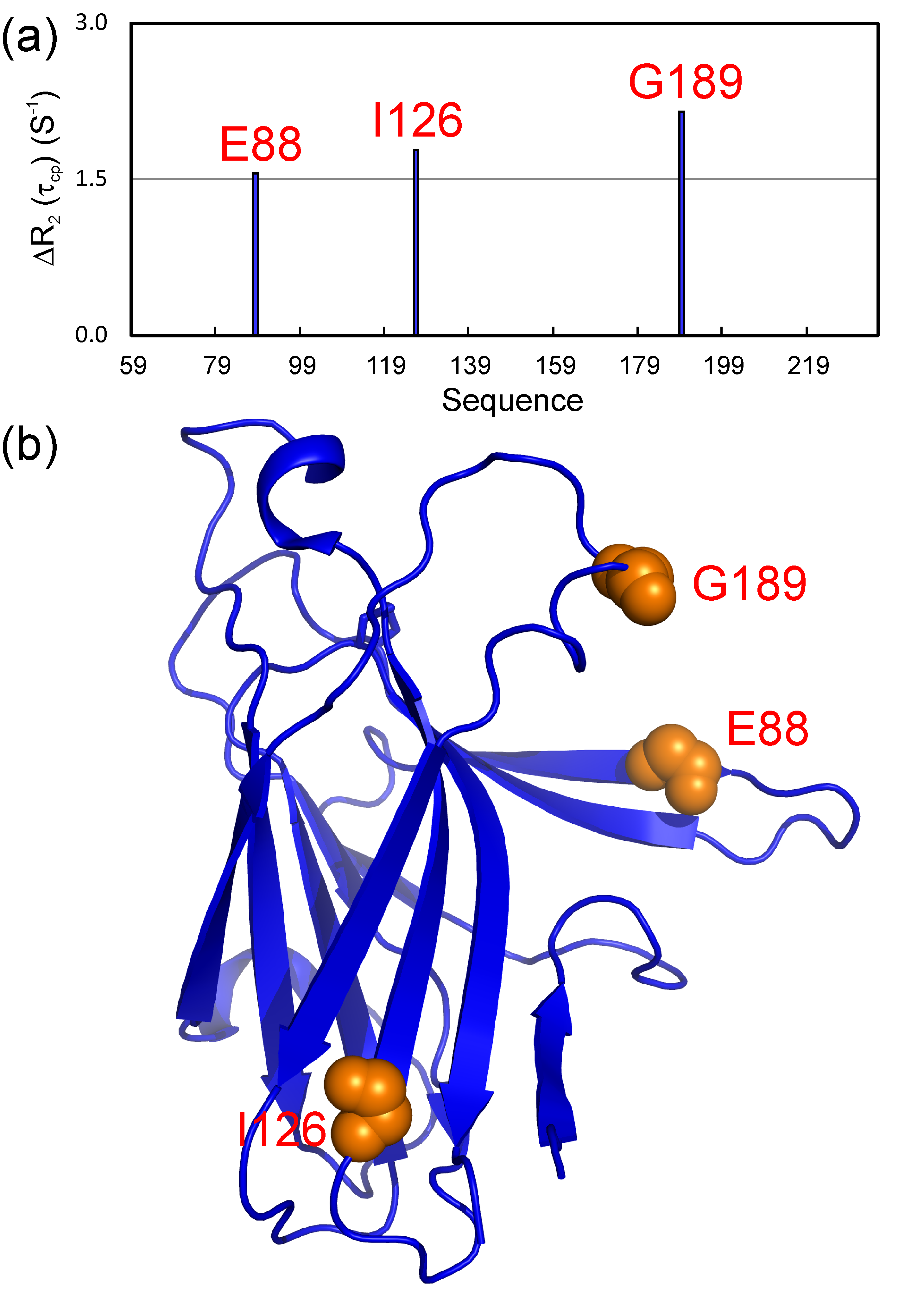

Supplement: Figure S2 — CPMG dispersion measurements reveal that the EphA5 LBD lacks global conformational exchanges in the µs-ms time scale. (a) Difference of effective transverse relaxation rate R2 (τcp) at 80 and 960 Hz. Only three residues have ΔR2 (τcp) >1.5 Hz, which indicates that only these residues have conformational exchanges on the µm-ms time scale. (b) EphA5 LBD with the three residues having ΔR2 (τcp) >1.5 Hz displayed as spheres. (TIF) [file pone.0074040.s002.tif]
