# Supplementary material for: Unique Structure and Dynamics of the EphA5 Ligand Binding Domain Mediate Its Binding Specificity as Revealed by X-ray Crystallography, NMR and MD Simulations
Source: PLoS One. 2013 Sep 24;8(9):e74040. doi: 10.1371/journal.pone.0074040 (PMC3782497; doi:10.1371/journal.pone.0074040)
Supplement: Table S1 — Crystallographic data and refinement statistics for the EphA5 LBD structure. (DOCX) [file pone.0074040.s003.docx]

**Table S1. Crystallographic data and refinement statistics for the EphA5 LBD structure**

|  | ***Data collection*** |  |
| --- | --- | --- |
|  | Wavelength (Å) | 1.5418 |
|  | Resolution range (Å) | 41.52 to 2.08 |
|  | Space group | C222(1) |
|  | ***Cell parameters*** |  |
|  | a, b, c (Å) | 55.04, 82.72, 81.17, |
|  | α, β, γ (°) | 90, 90, 90 |
|  | Observed Reflections | 10382 |
|  | Unique Reflections | 5385 |
|  | Redundancy | 23.12 |
|  | Completeness (%) | 90.60% |
|  | Overall (*I/σI)* | 1.6 |
|  | Rsym | 0.134 |
|  | ***Refinement*** |  |
|  | Resolution range (Å) | 20.68 to 2.6 |
|  | R_work_ | 0.2049 |
|  | No. of reflections | 4847 |
|  | R_free_ | 0.2824 |
|  | No. of reflections | 538 |
|  | RMSD bond lengths (Å) | 0.007 |
|  | RMSD bond angles (°) | 1.141 |
|  | ***Ramachandran Plot*** |  |
|  | Most favored region (%) | 76.8 |
|  | Additional allowed regions (%) | 23.2 |
|  | Generously allowed regions (%) | 0 |
|  | Disallowed regions (%) | 0 |
|  | ***B-factors*** |  |
|  | Protein | 31.347 |
|  | Water | 31.961 |

R_work_= Σ |Fobs - Fcalc|/ ΣFobs where Fcalc and Fobs are the calculated and observed structure factor amplitudes, respectively.

R_free_=as for Rwork, but for 10.3% of the total reflections chosen at random and omitted from refinement.
