# Supplementary material for: Unique Structure and Dynamics of the EphA5 Ligand Binding Domain Mediate Its Binding Specificity as Revealed by X-ray Crystallography, NMR and MD Simulations
Source: PLoS One. 2013 Sep 24;8(9):e74040. doi: 10.1371/journal.pone.0074040 (PMC3782497; doi:10.1371/journal.pone.0074040)
Supplement: Table S2 — Characteristics of the overall rotational diffusion of the EphA5 LBD. (DOCX) [file pone.0074040.s004.docx]

**Table S2. Characteristics of the overall rotational diffusion of the EphA5 LBD**

| **Protein** | $\boldsymbol{D}_{\boldsymbol{x}}$***** | $\boldsymbol{D}_{\boldsymbol{y}}$***** | $\boldsymbol{D}_{\boldsymbol{z}}$***** | $\boldsymbol{\phi}$ | $\boldsymbol{ϴ}$ | $\boldsymbol{\psi}$ | $\boldsymbol{\tau}_{\boldsymbol{c}}$ (ns) |
| --- | --- | --- | --- | --- | --- | --- | --- |
| **EphA5** | 0.83 ± 0.29 | 1.72 ± 0.46 | 2.21 ± 0.28 | 37 ± 17 | 72 ± 14 | 39 ± 17 | 12.2 |

*Principle values (in 10^7^s^-1^) of the rotational diffusion tensor. Ordered as *D_x_*$\leq$*D_y_*$\leq$*D_z_*.
